# Serum level of TNF-a, IL-1 and IL-6 and serum biomarkers after nutritional change in patients' ventilator-associated pneumonia

**DOI:** 10.5937/jomb0-56222

**Published:** 2025-08-21

**Authors:** Lu Peng, Wang Kun, Zheng Lihua, Li Dongliang, Zhang Jie, Su Ziqin

**Affiliations:** 1 The First Hospital of Hebei Medical University, Department of Intensive Care Unit I, Shijiazhuang, China

**Keywords:** TNF-a, IL-1 and IL-6, high-protein nutritional support, probiotics, critically ill patients, nutritional intervention, ventilator-associated pneumonia, TNF-a, IL-1 i IL-6, visokoproteinska nutritivna podrška, probiotici, kritično oboleli pacijenti, nutritivna intervencija, pneumonija povezana sa aspiratorom

## Abstract

**Background:**

This study aims to evaluate the effects of high-protein nutritional support (HPNS) combined with probiotics (Bifid Triple Viable Capsule) on the nutritional status, biochemical markers, and immune function in critically ill patients (CIPs) requiring mechanical ventilation, with a focus on preventing ventilator-associated pneumonia (VAP). The study explores how this intervention impacts serum albumin, prealbumin, and inflammatory cytokines (TNF-a, IL-1, IL-6), key nutritional and immune function indicators.

**Methods:**

This study included 86 critically ill patients requiring mechanical ventilation in the ICU. Participants were randomly assigned to either a research group (n=46) receiving HPNS combined with probiotics (Bifid Triple Viable Capsule) or a control group (n=40) receiving standard nutritional support. The primary outcomes included changes in serum albumin, prealbumin, and inflammatory cytokines (TNF-a, IL-1, IL-6) and the incidence of VAP The study duration was 3 weeks, and biochemical markers and clinical outcomes were assessed at baseline and post-intervention. The patients' body mass index (BMI) and body weight are calculated and compared. A spectrophotometer measures the content of serum protein. The prevalence of ventilator-associated pneumonia is analysed by sputum gram staining. The clinical symptoms of patients with ventilator-associated pneumonia during ICU are monitored. ELISA detects serum levels of inflammatory cytokines TNF-a, IL-1 and IL-6.

**Results:**

The research group demonstrated significant improvements in serum albumin and prealbumin levels and a lower TNF-a, IL-1, and IL-6 ratio than the control group (P< 0.05). The incidence of ventilator-associated pneumonia (VAP) was significantly lower in the research group (6.52%) compared to the control group (30%, P<0.05). Additionally, the research group showed higher BMI and body weight (P< 0.05), suggesting improved nutritional status following the intervention.

**Conclusions:**

High-protein nutritional support combined with probiotics can significantly raise the nutritional conditions of critically ill patients and effectively prevent ventilator-associated pneumonia. This intervention enhanced key biochemical markers, such as serum albumin, prealbumin, and the albumin/total protein ratio, all of which are important indicators of nutritional status. The improvement in these markers suggests that HPNS supports tissue repair and immune function, which are crucial for recovery in ICU patients. Additionally, the combination of HPNS and probiotics reduced serum levels of inflammatory cytokines (TNF-a, IL-1, IL-6), which are commonly elevated in critically ill patients and contribute to developing infections like VAP By regulating the inflammatory response, this intervention may help reduce the risk of infection and promote faster recovery. The results of this study highlight the potential clinical value of HPNS combined with probiotics for improving the management of critically ill patients in ICU settings.

## Abstract (sr)

**Uvod:**

Ova studija ima za cilj da proceni efekte visokoproteinske nutritivne podrške (HPNS) u kombinaciji sa probioticima (Bifid Triple Viable Capsule) na nutritivni status, biohemijske markere i imunološku funkciju kod kritično obolelih pacijenata (CIP) na mehaničkoj ventilaciji, sa posebnim fokusom na prevenciju pneumonije povezane sa respiratorom (VAP). Studija ispituje kako ova intervencija utiče na nivoe serumskog albumina, prealbumina i inflamatornih citokina (TNF-a, IL-1, IL-6), kljucnih pokazatelja nutritivnog statusa i imunološke funkcije.

**Metode:**

U studiju je uključeno 86 kritično obolelih pacije-nata koji zahtevaju mehaničku ventilaciju u jedinici intenzivne nege (ICU). Učesnici su nasumično raspoređeni u istraživačku grupu (n= 46), koja je primala HPNS u kombi-aciji sa probioticima (Bifid Triple Viable Capsule), i kontrolnu grupu (n= 40), koja je primala standardnu nutritivnu podršku. Primarni ishodi uključivali su promene u nivoima serumskog albumina, prealbumina i inflamatornih citokina (TNF-a, IL-1, IL-6), kao i učestalost VAP Trajanje studije bilo je tri nedelje, a biohemijski markeri i klinički ishodi procenjivani su na početku i nakon intervencije. Indeks telesne mase (BMI) i telesna masa pacijenata su izračunati i upoređeni. Sadržaj serumskih proteina meren je spektrofotometrom. Prevalenca pneumonije povezane sa ventilatorom analizirana je gramovim bojenjem sputuma. Klinički simptomi pacijenata sa VAP tokom boravka u ICU su praćeni. ELISA metodom su detektovani nivoi inflama-tornih citokina TNF-a, IL-1 i IL-6 u serumu.

**Rezultati:**

Grupa nad kojom je sprovođeno istraživanje je pokazala značajna poboljšanja u nivoima serumskog albu-mina i prealbumina, kao i niži odnos TNF-a, IL-1 i IL-6 u poređenju sa kontrolnom grupom (P< 0,05). Učestalost pneumonije povezane sa ventilatorom (VAP) bila je značaj-no niža u grupi nad kojom je sprovođeno istraživanje (6,52%) u poređenju sa kontrolnom grupom (30%, P<0,05). Takođe, grupa nad kojom je sprovođeno istraživanje je imala veće vrednosti BMI i telesne mase (P< 0,05), što ukazuje na poboljšan nutritivni status nakon intervencije.

**Zaključak:**

Visokoproteinska nutritivna podrška u kombinaciji sa probioticima može značajno poboljšati nutritivni status kritično obolelih pacijenata i efikasno smanjiti rizik od pneumonije povezane sa ventilatorom. Ova intervencija je poboljšala ključne biohemijske markere, poput serumskog albumina, prealbumina i odnosa albumin/ukupni pro-tein, koji su važni pokazatelji nutritivnog statusa. Poboljšanje ovih markera ukazuje na to da HPNS podržava reparaciju tkiva i imunološku funkciju, što je ključno za oporavak pacijenata u ICU. Pored toga, kombinacija HPNS i probiotika smanjila je nivoe inflamatornih citokina (TNF-a, IL-1, IL-6) u serumu, koji su često povišeni kod kritično obolelih pacijenata i doprinose razvoju infekcija poput VAP Regulišući inflamatorni odgovor, ova intervencija može pomoći u smanjenju rizika od infekcija i ubrzanju oporavka. Rezultati ove studije ističu potencijalnu kliničku vrednost HPNS u kombinaciji sa probioticima za unapređenje lečenja kritično obolelih pacijenata u ICU okruženju.

## Introduction

With the continuous progress of medical technology, the level of treatment in intensive care units (ICUs) is constantly improving. However, critically ill patients (CIPs) require long-term and sustained use of ventilators for life support due to severe diseases and trauma [1]. In this highly stressed and metabolic state, patients are prone to the risk of malnutrition, which seriously affects their recovery and prognosis. Meanwhile, ventilator-associated pneumonia (VAP), as a common and serious complication in the ICU, poses a considerable challenge to the treatment and rehabilitation process of CIPs. Therefore, exploring effective nutritional support strategies to raise the nutritional conditions of CIPs and prevent VAP has become an urgent issue in clinical research and medical practice. In this context, the high protein nutritional support intervention program combined with probiotics (Bifidobacterium Triple Viable Capsules) has attracted widespread attention [2] [3]. The highprotein nutritional support (HPNS) combined probiotic intervention has received significant attention in the clinical treatment of CIPs.

Protein is crucial to maintaining tissue repair and immune function in the body. In CIPs, the increase in metabolic rate and stress response leads to a relatively high demand for protein [4] [5]. HPNS, as one of the important means to improve nutritional status, can provide sufficient protein supply, maintain muscle mass and immune function, and help promote patient recovery. However, there is still some controversy over whether a single HPNS is sufficient to meet the needs of CIPs. Probiotics are microorganisms that are beneficial to the host and play an essential role in maintaining intestinal health and balance and regulating immune function [6] [7]. In recent years, probiotics have been increasingly valued in clinical applications, demonstrating potential benefits in preventing infections and maintaining intestinal microbiota balance [8] [9]. On the basis of HPNS, combined with probiotic intervention may help optimise the intestinal environment, promote protein absorption and utilisation, further improve the effectiveness of nutritional support, and thus improve the nutritional conditions of CIPs. This study aims to evaluate the biochemical effects of high-protein nutritional support (HPNS) combined with probiotics (Bifidobacterium Triple Viable Capsule) on nutritional conditions and immune function in critically ill patients (CIPs) requiring mechanical ventilation, with a focus on preventing ventilator-associated pneumonia (VAP). The study also explores how this combined intervention impacts key biochemical markers of nutrition (serum albumin, prealbumin) and inflammatory cytokines (TNF-α, IL-1, IL-6), which are critical for improving patient outcomes in ICU settings.

Theoretically, the findings contribute to the growing understanding of the gut-lung axis, which links gut health with lung function. By optimising the gut microbiota, HPNS combined with probiotics may help mitigate the risk of infections like VAP by improving nutritional status and immune function. These results highlight the importance of integrating nutritional support and gut microbiota regulation in ICU care, offering new insights into how these factors interact to enhance patient recovery.

From a practical perspective, this study suggests that incorporating HPNS and probiotics into ICU protocols could significantly improve nutritional outcomes while reducing the risk of infections like VAP The improvement observed in both nutritional conditions and immune responses supports the idea that this intervention could become a cornerstone of ICU care, helping critically ill patients recover more effectively and reducing complications associated with prolonged mechanical ventilation.

## Materials and methods

### General information

86 CIPs were recruited who needed continuous ventilator support in the ICU department of the central hospital from March 2023 to December 2024 as the research subjects. The patient's age ranges from 26 to 71 years, with an average age of 64.72±7.34. Among them, there are 48 male and 38 female patients. The patients are randomly broken into the research group (n = 46) and the control group (n=40). The research group will receive HPNS and intervention with probiotics (Bifidobacterium Triple Viable Capsules), while the control group will receive standard nutritional support. The research period is 3 months. Patients sign the protocol informed consent when included in the study.

Inclusion criteria: CIPs aged 18 years and above; Patients who require continuous ventilator support; Expected life >3 months; Normal intestinal function without obvious history of intestinal diseases or surgeries; Receiving HPNS and probiotic intervention without contraindications; The patient or their legal guardian is willing to sign an informed consent form.

Exclusion criteria: Patients who have not used a ventilator or whose estimated support time is less than 72 hours; Patients who have received HPNS or probiotic intervention; Patients with known allergies to high protein diets or probiotics; Patients with severe liver dysfunction or severe renal insufficiency; Patients with severe gastrointestinal diseases, such as acute pancreatitis and severe inflammatory bowel disease; Pregnant or lactating women; Other serious underlying diseases or complications, such as advanced cancer or severely infected patients; Patients who are unable to cooperate or participate in the research plan, such as those with intellectual disabilities or communication difficulties.

### Medical ethics issues

The Ethics Committee of the Central Hospital supports this study. Before written consent, all patients are informed of the complete study protocol information.

### Research procedure

Record the following demographic information: age, gender, weight, body mass index (BMI), serum protein content, and smoking and drinking habits. In terms of dietary intervention, the control group received enteral nutrition emulsion TPF (Rexen) (Hu- arui Pharmaceutical Co., Ltd., national drug approval number H20040188, specification: 500 mL/bottle), and nutritional support is provided with whey protein powder (Xi'an Libang Clinical Nutrition Co., Ltd., production license number QS610127016001, specification: 300 g/can) according to the total protein intake standard of 1.5-2.0 g/cal/d. On the ground of the control group, the research group developed an individualised high-protein nutrition plan based on the patient's weight, disease status, and energy needs. It intervened with probiotics (Bifidobacterium Triple Viable Capsule). HPNS requires calculating the total daily energy supply based on the patient's ideal body mass, i.e. daily non-protein energy (Kcal) = 25 (Kcal) x ideal body mass (kg), ideal body mass= height (cm) -105. Probiotics are Bifidobacterium Triple Viable Capsules. The research period is 3 months, and the infection rate, clinical symptoms, and nutritional indicators of VAP are compared.

### Blood sample collection and Inflammatory factor analysis

After fasting for 8 hours, the patient's blood samples are collected using a serum separation tube and left at room temperature for at least 30 minutes. Then, the sample is centrifuged at 3500 rpm for 10 minutes, and the serum is divided into 2 mL tubes and stored at -80°C for analysis. An ELISA detection kit purchased from eBioscience™ (Thermo Fisher, Waltham, Massachusetts, USA) is used to measure tumour necrosis factor-α (TNF-α), interleukin-1 (IL-1), and interleukin-6 (IL-6). With the manufacturer's instructions, a standard curve is constructed to measure the concentration of various cytokines.

### Sputum staining and microbial culture

Medical staff obtained 2 mL of sputum samples from the patient's trachea or bronchus using Gram staining and fast staining. Then, the sputum samples are applied separately to the appropriate culture media. Then, the culture medium is placed in a constant temperature incubator (under constant temperature conditions of 35-37°C) for cultivation. From 24 to 48 hours of observation, laboratory technicians will conduct bacterial identification to determine the types and characteristics of bacteria.

### Detection of serum protein content

Serum protein content is detected using a spectrophotometer. As mentioned, blood samples are collected from patients and centrifuged to obtain serum. The serum sample size is approximately 1 millilitre. The spectrophotometer is opened, and the parameters are adjusted to 280 nanometers (nm). After calibration, the prepared serum sample is added to the sample pool of the spectrophotometer. The absorbance value of the sample is recorded, and then the content of the target protein in the sample is calculated based on the absorbance value [10].

### Statistical analysis

SPSS version 25.0 is utilised to analyse the data. Frequency and percentage are utilised to define descriptive features of patients. Mann-Whitney U test is applied to determine the difference between two types of independent variables. The chi-square test is used to classify variables for analysis. All analyses using the double-tailed test have a significance level* P *value of less than 0.05.

## Results

### General data analysis

Statistical comparison of clinical data showed that the control group had a male-to-female ratio of 22:18, an average age of 63.58±5.21 years, an average BMI of 20.37±1.88 kg/m^2^, an average weight of 65.37±11.54 kg. Biochemical analysis denoted that serum albumin levels were 32.27±5.26 g/L, prealbumin levels were 163.24±10.33 mg/L, and haemoglobin levels were 124.29±13.08 g/L, with 15 cases smoking and 7 cases drinking. The male-to-female ratio of the research group was 26:20, with an average age of 65.42±4.35 years old, an average BMI of 20.15±2.02 kg/m^2^, and an average weight of 63.71 ±12.36 kg. Biochemical analysis denoted that the serum albumin level was 33.85±5.49 g/L, prealbumin was 158.55±11.24 mg/L, and haemoglobin was 119.61 ±12.58 g/L, with 17 cases smoking and 10 cases drinking. The comparison of general information is with *P*>0.05, as expressed in Table 1.

**Table 1:** General data analysis.

Items	Control group (n=40)	Research group (n=46)	T value/χ^2^ value	P value
Gender (male: female)	22:18	26:20	3.119	0.492
Age (years)	63.58±5.21	65.42±4.35	5.206	0.215
BMI (kg/m^2^)	20.37±1.88	20.15±2.02	3.418	0.306
Weight (kg)	65.37 ±11.54	63.71 ±12.36	3.965	0.115
Serum albumin (g/L)	32.27±5.26	33.85±5.49	2.773	0.268
Prealbumin (mg/L)	163.24±10.33	158.55±11.24	1.431	0.571
Hemoglobin (g/L)	124.29±13.08	119.61 ±12.58	2.809	0.435
Smoke	15 (37.50%)	17 (36.95%)	4.615	0.829
Drink	7 (17.50%)	10 (21.73%)	3.077	0.224

### Analysis of changes in body weight and BMI

Patients' BMI and body weight were calculated and compared, and data analysis showed that the weight and BMI of the control group patients were lower than those of the research group (*P*<0.05) (Table 2).

**Table 2:** Analysis of changes in patient weight and BMI.

Group	Weight (kg)	BMI (kg/m^2^)
Control group (n=40)	61.54±6.25	18.51 ±1.36
Research group (n=46)	72.67±7.39	21.49±1.68
*t* value	10.558	11.302
*P* value	0.015	0.026

### Analysis of serum protein content

The serum protein content of patients was measured using a spectrophotometer, and the levels of serum albumin, prealbumin, and serum albumin/total protein ratio in the control group were lower than those in the research group (*P*<0.05) (Table 3).

**Table 3:** Analysis of serum protein content in patients.

Group	Serum albumin (g/L)	Prealbumin (mg/L)	Serum albumin/total protein
Control group (n=40)	28.24±4.47	155.39±9.66	0.53±0.08
Research group (n=46)	35.37 ±3.25	194.26±10.34	0.67±0.10
*t* value	9.521	11.304	13.586
*P* value	0.014	0.025	0.008

### Pneumonia Infection rate statistics

The prevalence of ventilator-acquired pneumonia in patients was detected through sputum microbial culture. In the control group, there were 6 cases of Gram-negative bacterial infections, 4 cases of Gram-positive bacterial infections, and 2 cases of other bacterial infections, with a pneumonia prevalence rate of 30%. The research group had 2 Gram-negative bacterial infections and 1 Gram-positive bacterial infection, with a pneumonia prevalence rate of 6.52%. The infection rate of VAP in the control group was higher than that in the research group (*P*<0.05) (Table 4).

**Table 4:** Statistics of pneumonia infection rate.

Group	Gram-negative bacteria (%)	Gram-positive bacteria (%)	Others (%)	Total (%)
Control group (n=40)	6 (15.00%)	4 (10.00%)	2 (5.00%)	12 (30.00%)
Research group (n=46)	2 (4.37%)	1 (2.17%)	0 (%)	3 (6.52%)
*t* value	9.557	11.834	3.016	14.227
*P* value	0.016	0.028	0.106	0.003

### Clinical symptom statistics of VAP

The clinical symptoms VAP patients experience during ICU were monitored and counted, such as fever, cough, rapid breathing, and lung signs such as rales. There were 5 cases of fever, 8 cases of cough, 3 cases of rapid breathing, and 4 cases of rale in the control group, with a total incidence rate of 50%. There were 2 cases of fever, 3 cases of cough, and 1 case of rale in the experimental group, with a total incidence rate of 13.04%. The incidence of clinical symptoms of VAP in the control group was higher than that in the research group (*P*<0.05) (Table 5).

**Table 5:** Clinical symptom statistics of VAP

Group	Fever	Cough	Rapid breathing	Rale	Total (%)
Control group (n=40)	5 (12.50%)	8 (20.00%)	3 (7.50%)	4 (10.00%)	20 (50.00%)
Research group (n=46)	2 (4.34%)	3 (6.52%)	0 (0.00%)	1 (2.17%)	6 (13.04%)
*t* value	9.411	8.536	10.532	12.405	15.624
*P* value	0.025	0.037	0.018	0.045	0.003

### Analysis of serum Inflammatory factors

ELISA was applied to detect the degrees of serum Inflammatory factors TNF-α, IL-1, and IL-6 In patients. Thees In the research group were lower than those In the control group (*P*<0.05) (Table 6).

**Table 6:** Analysis of serum inflammatory factors.

Group	TNF-α (pg/mL)		IL-1 (pg/mL)		IL-6 (pg/mL)	
	Before treatment	After treatment	Before treatment	After treatment	Before treatment	After treatment
Control qroup<br>(n=40)	70.78±11.52	48.29±6.18	28.12±4.12	17.36±3.44	46.63±5.28	37.34±4.52
Research group<br>(n=46)	67.88±12.75	22.35±4.66	27.51 ±3.65	8.18±2.05	48.10±6.16	15.29±3.57
*t* value	1.250	11.662	1.024	9.035	1.267	13.726
*P*-value	0.167	0.012	0.216	0.006	0.139	0.027

## Discussion

CIPs require continuous use of ventilators for life support due to severe Illness or trauma [11]. This study found that HPNS combined with probiotics significantly Improved the nutritional status of critically ill patients (CIPs), as evidenced by Increased body weight, BMI, and serum protein levels (albumin and prealbumin). These results align with existing literature suggesting that protein supplementation Is critical In maintaining Immune function and facilitating recovery In CIPs. Given the heightened metabolic demands In ICU patients, particularly those on mechanical ventilation, maintaining adequate nutrition Is essential to support tissue repair and Immune defence mechanisms. Previous studies have shown that protein supplementation helps preserve muscle mass, reduce catabolism, and Improve Immune response, all of which are critical In ICU settings [12]. The Improvement In serum protein levels, mainly albumin, reflects the beneficial Impact of this Intervention on protein nutrition, which Is vital for recovery In critically ill patients. In the ICU, these patients' body metabolism Is highly stressed and metabolic, Increasing the body's demand for energy and nutrients. However, due to the Impact of diseases and the limitations of medical Interventions, CIPs often face the risk of malnutrition. Malnutrition Is widespread In CIPs and may harm their recovery and prognosis [13]. Malnutrition may decrease the body's Immune function and susceptibility to Infection and delay wound healing and tissue repair. In addition to reducing Infection rates, the significant reduction In proinflammatory cytokines (TNF-α, IL-1, IL-6) In the research group provides further evidence of the efficacy of the combined HPNS and probiotic Intervention.

Inflammation Is a key player In the pathogenesis of VAF) and these cytokines are central mediators In the Inflammatory response. TNF-α (Tumor Necrosis Factor-alpha) Is a potent pro-inflammatory cytokine that Induces fever, Increases vascular permeability, and promotes Immune cell recruitment to sites of Infection. In critically ill patients, elevated levels of TNF-α are associated with poor outcomes, Including Increased mortality and complications such as VAP IL-1 (lnterleukln-1) also plays a central role In Initiating and amplifying the Inflammatory response by stimulating the production of other cytokines and acute-phase proteins. Elevated IL-1 levels are linked to systemic Inflammatory response syndrome (SIRS) and sepsis, both of which are prevalent In ICU patients and contribute to VAP development. IL-6 Is another Important cytokine Involved In Inflammation, acting as a pro-inflammatory and Immunoregulatory molecule. High IL-6 levels In critically ill patients are Indicative of ongoing Inflammation and are associated with poor clinical outcomes, Including prolonged ICU stays and Increased risk of Infections like VAP [14].

The ability of probiotics to reduce the levels of these cytokines suggests that the combined Intervention of HPNS and probiotics may help modulate the systemic Inflammatory response, preventing excessive Inflammation that contributes to VAP development. By reducing cytokine levels, the Intervention may Improve the patient's Immune response, reduce tissue damage, and limit the progression of Infection [15].

Studies suggest that probiotics can suppress excessive cytokine release, helping to maintain Immune homeostasis In critically ill patients. The reduction In Inflammatory markers observed In the study Indicates that HPNS combined with probiotics can help control the systemic Inflammatory response In ICU patients, which may contribute to the reduced Incidence of VAP and Improved clinical outcomes [16]. In addition, malnutrition may also lead to muscle depletion and abnormal protein metabolism, further affecting patients' life quality and recovery speed. In addition to HPNS, probiotics provide significant benefits by modulating gut microbiota, enhancing intestinal barrier function, and regulating systemic immune responses. This is particularly important in ICU patients, who often experience gut dysbiosis due to stress, mechanical ventilation, and antibiotic use. Results showing reduced infection rates and better clinical outcomes (lower incidence of VAP symptoms and inflammatory markers) suggest that probiotics play a critical role in improving gut health, which is linked to preventing infections like VAP. Probiotics restore a healthy balance of gut microbiota, thereby supporting nutrient absorption, boosting immune response, and reducing the risk of infections. These findings suggest that probiotics can help regulate the gut-lung axis, preventing the translocation of bacteria from the gut to the lungs, which is a key factor in the development of VAP [17]. VAP is a common and serious complication in CIPs. Due to prolonged use of ventilators, patients' airways are prone to contamination and increase the risk of bacterial and viral infections [18] [19]. The reduced incidence of VAP in the research group (6.52%) compared to the control group (30%) underscores the efficacy of HPNS combined with probiotics in preventing VAP This significant difference supports the growing body of evidence indicating that nutrition and gut health interventions can reduce infection rates. The reduction in VAP incidence can be attributed to the improvement in nutritional status and immune function and the positive effects of probiotics on gut health.

Furthermore, the reduction in clinical symptoms such as fever, cough, rapid breathing, and rales in the research group suggests that the intervention reduced infection and mitigated the severity of clinical symptoms, improving the overall quality of life and recovery speed for ICU patients. VAP not only prolongs the hospitalisation time of patients, increases medical costs, but may also lead to serious complications and even endanger life [20]. Therefore, preventing VAP in the ICU is crucial for improving the prognosis of CIPs and reducing medical costs [21]. To promote the nutritional conditions of CIPs and prevent VAP, HPNS combined with probiotic intervention has become a highly focused strategy. HPNS can provide sufficient nutrients to maintain the body's immune function and muscle mass [22]. The supplementation of probiotics can help regulate the intestinal microbiota, enhance intestinal barrier function, and reduce the risk of infection.

This study recruited 86 CIPs who continued to use ventilator support and found that HPNS combined with probiotics significantly improved the nutritional status of CIPs. Under the support of high-protein nutrition, the weight and BMI of CIPs significantly increased, and the serum albumin, serum prealbumin, and serum albumin/total protein ratio significantly increased, indicating an improvement in the patient's protein nutrition level. At the same time, the supplementation of probiotics helped to maintain the balance of intestinal microbiota, promote the absorption of nutrients, and regulate immune function. It may also have a particular role in preventing complications such as VAP.

Malnutrition is an ordinary problem among CIPs, and HPNS is an important means to improve nutritional conditions [23]. Protein is an important component of tissue repair and immune function, while CIPs have a relatively high demand for protein due to increased metabolic rate and stress response. By providing sufficient protein, muscle mass and immune function can be maintained, which helps promote patient recovery [24]. Probiotics are beneficial microorganisms that can regulate intestinal microbiota balance, enhance intestinal barrier function, inhibit the growth of harmful bacteria, and promote nutrient absorption [25] [26]. Based on high protein nutritional support, adding probiotic interventions may further optimise the intestinal environment, promote protein absorption and utilisation, and enhance the effectiveness of nutritional support. Body weight and BMI are important indicators for measuring nutritional status. The increase in weight and BMI of patients in the research group under the intervention of HPNS combined with probiotics reflected an improvement in their nutritional status. An increase in weight may indicate that the patient was beginning to regain tissue quality and level, and an increase in BMI also indicated an improvement in the balance between weight and height, which positively impacted the patient's recovery and disease prognosis. Serum albumin and prealbumin are important representatives of proteins in the body and are also commonly used indicators to evaluate protein nutritional status [27] [28]. This study found that under the intervention of HPNS combined with probiotics, the degrees of serum albumin and prealbumin in the research group patients increased, indicating an improvement in protein supply. In addition, the increase in serum albumin/total protein ratio also reflected the increase in the proportion of hemorrhagic albumin in total protein, which is usually a sensitive indicator to measure the protein nutritional status of patients. Through a 3-month dietary intervention, it was found that the intervention of HPNS combined with probiotics was effective in improving the nutritional status of CIPs. Providing sufficient protein supply and optimising the gut microbiota helped improve patients' nutritional levels and enhance immune function.

HPNS combined with probiotics has shown significant potential effects in preventing VAR Statistics denoted that the occurrence of VAP in the control group was as high as 30%, while in the research group, it was only 6.52%. This huge difference indicated that HPNS combined with probiotics might have a specific protective effect on reducing the occurrence of VAR In addition, the clinical symptom incidence of VAP was significantly reduced in the research group of patients under the intervention of HPNS combined with probiotics. The clinical symptoms of fever, cough, rapid breathing, and rales were significantly reduced compared to the control group, indicating that HPNS combined with probiotics might help alleviate the clinical manifestations of VAP and improve patients' quality of life and rehabilitation effects. The degree of inflammatory factors was also key in preventing VAP [29] [30]. This study found that the degrees of serum inflammatory factors in the research group were lower than those in the control group. This might mean that HPNS combined with probiotic intervention could reduce the release of inflammatory factors by regulating the inflammatory response, thereby reducing the occurrence of VAR This provided some clues to deeply understand the mechanism of action of HPNS combined with probiotics. The above results suggested that HPNS combined with probiotics had potential positive effects in improving the nutritional conditions of CIPs and preventing VAR This intervention might protect CIPs by optimising nutrient supply, regulating intestinal microbiota balance, and inhibiting inflammatory reactions. Despite the promising results, there are several limitations to this study. The relatively short study duration (3 weeks) limits the ability to assess the long-term effects of HPNS combined with probiotics. Further studies with more extended follow-up periods would help determine whether the benefits of this intervention persist over time.

Additionally, the study was conducted at a single centre with a relatively small sample size, which may limit the generalizability of the findings. Therefore, multicenter trials with larger sample sizes must validate these results. Future research should also explore the mechanisms behind the effects of probiotics on gut microbiota and the immune system. Specifically, studies examining how probiotics modulate the gut-lung axis and their impact on intestinal permeability and immune response in ICU patients would provide valuable insights into the underlying processes contributing to the observed benefits.

## Conclusion

In summary, the results of this study demonstrate that HPNS combined with probiotics has a significant improvement effect on the nutritional conditions of CIPs and can effectively prevent the happening of VAR This intervention may play a role by improving patients' nutritional levels and regulating inflammatory responses. It also has potential clinical application value in managing CIPs in the ICU.

## Dodatak

### Funding

The Scientific research fund project of Hebei Provincial Health Commission, (No. 20190469) supports the research.

### Conflict of interest statement

All the authors declare that they have no conflict of interest in this work.
